# Sublobectomy is a safe alternative for localized cavitary pulmonary tuberculosis

**DOI:** 10.1186/s13019-021-01401-5

**Published:** 2021-03-17

**Authors:** Yong Yang, Shaojun Zhang, Zhengwei Dong, Yong Xu, Xuefei Hu, Gening Jiang, Lin Fan, Liang Duan

**Affiliations:** 1 Department of Thoracic Surgery, Shanghai Pulmonary Hospital, Tongji University School of Medicine, No. 507 Zhengmin Road, Shanghai, 200433 China; 2 Clinic and Research Center of Tuberculosis, Shanghai Key Lab of Tuberculosis, Shanghai Pulmonary Hospital, Tongji University School of Medicine, No. 507 Zhengmin Road, Shanghai, 200433 China; 3 Department of Pathology, Shanghai Pulmonary Hospital, Tongji University School of Medicine, Shanghai, 200433 China

**Keywords:** Pulmonary tuberculosis, Cavity, Lobectomy, Sublobectomy, Complication, Prognosis

## Abstract

**Objective:**

Surgical resection plays an essential role in the treatment of Pulmonary Tuberculosis (PTB). There are few reports comparing lobectomy and sublobectomy for pulmonary TB with cavity. To compare the advantages between lobectomy and sublobectomy for localized cavitory PTB, we performed a single-institution cross sectional cohort study of the surgical patients.

**Methods:**

We consecutively included 203 patients undergoing lobectomy or sublobectomy surgery for localized cavitary PTB. All patients were followed up, recorded and compared their surgical complication, outcome and associated characteristics.

**Results:**

Both groups had similar outcomes after follow up for 13.1 ± 12.1 months, however, sublobectomy group suffered fewer intraoperative blood losses, shorter length of stay, and fewer operative complications than lobectomy group (*P* <  0.05). Both groups obtained satisfactory outcome with postoperatively medicated for similar period of time and few relapse (*P* > 0.05).

**Conclusion:**

Both sublobectomy and lobectomy resection were effective ways for cavitary PTB with surgical indications. If adequate anti-TB chemotherapy had been guaranteed, sublobectomy is able to be recommended due to more lung parenchyma retain, faster recover, and fewer postoperative complications.

## Introduction

Tuberculosis (TB) is still a major public health threat that is caused by the *Mycobacterium tuberculosis* (MTB) complex [1]. According to the update investigation issued from World Health Organization, there were 10 million new TB cases and 1.45 million deaths from TB in 2018 [2]. Pulmonary cavitation is one of the features in pulmonary TB (PTB) and one of the most frequently observed in clinic. It accounts for more than 40% of adults with PTB [3, 4]. Multiple laboratory tests showed that cavitation was related to higher bacterial load in the sputum [5, 6]. The researchers compared the bacillary load in lung sections from resected lung tissues of PTB patients, and found that the bacillary load in the cavity walls was 10^5^ times higher than that in the caseous necrosis [7]. The presence of cavitary disease is associated with treatment failure and relapse of PTB [8]. Therefore, effective management of cavity is important for the treatment of PTB.

Before the availability of chemotherapy for TB, operation was the indispensable choice for the treatment, with the development of anti-TB drugs, antibiotic treatment became the main therapy for TB [9]. As drug-resistant TB (DR-TB) is a new threat to the patients, lung resection is increasingly being explored as an option for patients with DR-TB. Either lobectomy, bilobectomy, pneumonectomy, or staged lobectomies were adopted for localized bilateral disease. Wedge or segmental resections are usually avoided because of the increased risk of bronchopleural fistula [10]. However, following the improvement of surgical techniques and anti-TB chemotherapy, sublobectomy may be considerable for PTB patients with well-localized cavity. Recently, WHO consolidated guidelines on DR-TB indicated that in patients with RR-TB or MDR-TB, elective partial lung resection (lobectomy or wedge resection) may be used alongside a recommended MDR-TB regimen [11]. In this study, we aimed to compare the clinical effect of sublobectomy and lobectomy in patients with cavitary PTB.

## Materials and methods

### Patient enrollment

Between February 2012 and June 2018, the medical records of 1535 patients who underwent PTB resection were cross sectional reviewed. Among them, 457 patients with localized cavitary PTB received propensity score matching for age, lesion size, and preoperative chemotherapy time, and 203 patients were finally enrolled. One hundred twenty-nine patients received lobectomy and 74 received sublobectomy. The included criteria were as followed: patients who suffered from localized cavitary PTB and had indications for surgical resections as adjunct to chemotherapy, or with cavity which were difficult to be treated with drug-resistant TB or thick wall cavernous TB with being difficult to close with chemotherapy alone, and lesions were confined to one lobe with a localized cavity that can be recognized by imaging, and lesions can be resected with the expectation of adequate cardiopulmonary reserve after operation, and localized unilateral lesion together with the satellite lesions within 1 or 2 segments of the same lobe, and patients who finished all the course of treatment and had intact record of follow up at clinics; all included patients underwent PTB resection surgery with sublobectomy or lobectomy; Those who suffered from systemic or diffused PTB, underwent concomitant decortication or thoracoplasty, bronchoscopy for blood clot evacuation, undiagnosed before operation, failed to receive preoperative anti-TB medication, or lost to be followed-up were excluded.

Preoperative evaluation included history and clinical examination to address any comorbidity, especially diabetes, anemia, underlying lung disease, and hypoalbuminemia based on the performance of chest radiography, computed tomography, and pulmonary function tests and echocardiograms to exclude pulmonary artery hypertension. The treatment strategy was discussed between tuberculosis physicians and thoracic surgeons before operation. Sputum acid-fast stain and culture were routinely examined before and after surgery. Informed consent was waived because the study was retrospective. Clinical information was collected on age, sex, the ultimate surgical approach and procedures, operative time, blood loss, length of hospital stay, complication, chemotherapy, treatment outcome, and follow-up duration. The regimen of anti-TB medications was directed according to the guideline of WHO [12]. All included patients were followed throughout the surgical and chemical treatment. This study was approved by the ethics committee of Shanghai Pulmonary Hospital, and all the subjects had signed informed consent.

### Operation techniques

**Fig. 1:**
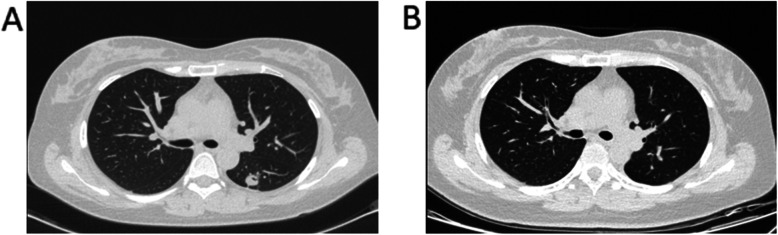
Localized cavitary pulmonary TB received sublobectomy and follow-up for 6 months. **a**, preoperation. **b**, post-operation

All the surgeries were done by thoracic surgeons with well experience of TB surgery. A lobectomy is defined as a procedure requiring dissection and ligation of vascular and bronchial structure. A sublobectomy is defined as the extent of resection less than a lobe either requiring dissection of sectional vascular and bronchus or not (Fig. 1), including segmentectomy and wedge resection [13]. The patient was placed in the full lateral decubitus position under general anesthesia with selective one-lung ventilation. The operations began with VATS may convert to open surgery on the condition of the dense adhesion in the pleural space, the lymph node around hilar structures, or huge bleeding precluded VATS. A utility thoracotomy of 3 to 5 cm at the fourth or fifth intercostal space was created and protected with a wound retractor. Specially, an incision on the fourth or fifth intercostal space of the anterior axillary line on both sides was selected for the upper lobe segmentectomy or lobectomy, while an incision on the fifth intercostal space of the anterior axillary line on both sides was chose for the middle or lower lobe segmentectomy or lobectomy. An electrocautery or ultrasound scalpel (Harmonic ACE1; Johnson & Johnson, Somerville, NJ) was used for pleural adhesiolysis and tissue separation. The major vascular branches were transected with endostaplers (Endo GIA 30-mm Articulating Vascular Reload, Covidien, Dublin, Ireland), whereas minor branches were treated by ligation, titanium clip (WECK Hem-o-lok, Telelex, Morrisville, NC, USA) or ultrasonic scalpel according to the diameter of the vessel. The lobar or segment bronchus was closed with endoscopic stapler. No muscle flap was used to reinforce bronchial closures. After the operation, one or two #28 chest tubes were placed through the incision for drainage. All the satellite lesions were completed removed by the operation. The pathology showed no tuberculosis residue on the cutting edge.

### Outcome evaluation

The cure was considered as the completion of treatment and at least three consecutive negative cultures from sputum and having no active lesions in lungs at the time of completed chemotherapy, the treatment success was define as patients getting cured added with the patients with less than three sputum negative cultures while completed all course of chemotherapy without active TB lesions after operations. Treatment failure was defined as culture positive or sputum conversion positive at the end of chemotherapy. Relapse was defined as the recurrence of positive smear or culture after the achievement of a cure. Short term and long term surgical complications were evaluated after operation.

### Statistical analysis

Patients were divided into two groups as received lobectomy or sublobectomy. The propensity score matching was used to balance the distributions of measured potentially confounding covariates for patients between groups. Continuous variables between these two groups (age, percentage of preoperative positive sputum culture, operative time, blood loss, length of hospital stay, duration of preoperative and postoperative medication, and postoperative follow-up) were compared using Mann-Whitney U test, whereas noncontinuous variables (sex, procedures, complication, and treatment result) were compared using a χ^2^ test. *P* <  0.05 was considered as statistical significance.

## Results

### Clinical characteristics

The preoperative demographics and variables of patients were shown in Table 1. The distribution of sex, age, and disease type were not significantly different. In the lobectomy group, there were 99 males and 30 females, with mean age of 50.1 ± 12.6 years. Patients had received a median of 5.8 ± 3.1 months of anti-TB drugs before the surgery. Major preoperative clinical manifestations contained coughing in 22 patients, chest pain in 11, expectoration in 13, haemoptysis in 10, chest distress in 4, and fever in 7. Preoperative positive sputum culture was observed in 16 patients. Except 9 lesions occupied two segments, the left 122 lesions were located in one segment. In the sublobectomy group, including 29 cases of segmentectomy and 47 cases of wedge resection, there were 59 males and 15 females with average age of 47.2 ± 11.7 years. Patients had received a median of 5.6 ± 2.7 months of anti-TB medical treatment before the surgery. The sublobectomy group had a similar length of anti-TB medication duration before surgery compared with the lobectomy group (*P* > 0.05, Table 1). Major preoperative clinical manifestations contained coughing in 18 patients, chest pain in 8, expectoration in 12, haemoptysis in 5, chest distress in 4, and fever in 3. Two cases suffered from positive sputum before surgery. There were 5 TB lesions located in two segments and 71 cases in only one segment.

**Table 1:** Preoperative characteristics of patients with cavitary pulmonary TB

Variable	Lobectomy (*n* = 129)	Sublobectomy (*n* = 74)	*P* value
**Gender**			
Male	99	59	0.62
Female	30	15	
**Age** (y, mean ± SD)	50.1 ± 12.6	47.5 ± 11.7	0.14
**Disease type**			
Primary treated	81	52	0.28
Retreated	48	22	
Culture positive in sputum (N)	9	2	0.19
Chemotherapy time (months, mean ± SD)	5.8 ± 3.0	5.6 ± 2.7	0.20
Multidrug resistance (N)	11	1	0.04*
Lesion diameter (cm, mean ± SD)	2.45 ± 0.90	2.12 ± 0.69	0.13
Cavity diameter (cm, mean ± SD)	1.01 ± 0.73	0.58 ± 0.49	< 0.001*
**Symptoms**			
Cough	22	18	0.21
Chest pain	11	8	0.59
Expectoration	13	12	0.20
Haemoptysis	10	5	0.79
Chest distress	4	4	0.41
Fever	7	3	0.66

*P < 0.05, sublobectomy group vs. lobectomy group

### Operation comparison

In the lobectomy group, 99 patients received VATS and 30 received thoracotomy. On the other hand, only 3 cases in the sublobectomy group received open surgery directly. The sublobectomy group had a significantly shorter operation time and fewer intraoperative blood losses than the lobectomy group (*P* <  0.01). Moreover, the rate of conversions to thoracotomy was similar between the two groups (*P* > 0.05). For postoperative recovery, the length of stay (LOS) in the lobectomy group was significantly longer than that of sublobectomy group (*P* <  0.05, Table 2).

**Table 2:** Operation comparison between two groups

Variable	Lobectomy (*n* = 129)	Sublobectomy (*n* = 74)	*P* value
Operation time, min	134.9 ± 57.7	107.9 ± 46.4	< 0.001*
Intraoperative blood loss, ml	137.6 ± 234.2	62.0 ± 51.5	0.007*
Conversions to thoracotomy, N	3	3	0.48
Length of stay	7.1 ± 14.6	4.3 ± 2.2	0.037*

*P < 0.05, sublobectomy group vs. lobectomy group

### Complications

There were 21 short term surgical complications in the lobectomy group and only 5 in the sublobectomy group (Table 4). In the lobectomy group, 7 cases suffered from increased amount of drainage and received blood transfusion. Six patients exhibited sustained air leak and had to take the tube for a long time. Three patients presented hemothorax and were controlled with the thoracoscopic approach. Two cases were complicated with chylothorax and managed with reoperation for thoracic duct ligation. One patient had pulmonary infection and empyema, which was controlled by drainage and anti-infection treatments. In addition, stress asthma and angina were observed in one case, respectively. On the contrary, only 2 cases of increased amount of drainage, 2 cases of sustained air leak, and 1 case of hemothorax were found in two patients in the sublobectomy group. The short term complication rate in the sublobectomy group was significantly lower than that in the lobectomy group (*P* < 0.05, Table 3).

**Table 3:** Postoperative short term complication comparison between two groups

Variable, N	Lobectomy (*n* = 129)	Sublobectomy (*n* = 74)
Increased amount of drainage	7	2
Sustained air leak	6	2
Hemothorax	3	1
Chylothorax	2	0
pulmonary infection and empyema	1	0
Stress asthma	1	0
Angina	1	0

### Outcomes

Both of two groups exhibited satisfied therapeutic effects without long term postoperative complication. During followed-up after surgery for 13.1 ± 12.1 months, the cure rate reached 98.5% despite of three cases relapses (Table 4). After operation, the patients in the lobectomy group received postoperative anti-TB medication for 7.7 ± 3.8 months, and the total medication duration reached 13.5 ± 10.2 months. In the lobectomy group, the patients were followed-up for a mean of 12.9 ± 11.7 months after operation, and most of the patients were cured without relapse. Three cases relapsed during follow-up, including one case was confirmed by pulmonary needle biopsy and two cases exhibited positive culture in sputum. All the patients with positive culture in sputum before operation obtained negative culture results during the postoperative follow-up. In the sublobectomy group, the patients received medication for 6.5 ± 2.9 months after surgery, and the total mean medication duration was 9.4 ± 9.4 months. The patients were followed-up for 14.1 ± 14.1 months and none of them exhibited relapse. There were 11 cases of MDR-TB in the lobectomy group and 1 MDR-TB patient in the sublobectomy group, only 1 patient relapsed in the lobectomy group, who received 6-month medication after surgery.

**Table 4:** Treatment outcome comparison between two groups

Variable (months)	Lobectomy (*n* = 129)	Sublobectomy (*n* = 74)	*P* value
Cure rate	97.6%	100%	0.18
Relapse rate	2.4%	0%	0.18
Long term surgery complication	0	0	–
Postoperative Culture positive in sputum, N	3	0	0.19
Postoperative medication duration	7.7 ± 3.8	6.5 ± 2.9	0.14
Medication duration	13.5 ± 10.2	9.4 ± 9.4	0.06
Follow-up	12.9 ± 11.7	14.1 ± 14.1	0.49

## Discussion

TB cavity is caused by the large load of tubercle bacillus damaging local lung tissue, leading to caseous necrosis. The lesion dissolves and perforates the bronchus, and the air enters to form the cavity. Pulmonary cavitation is one of the markers of PTB and is responsible for delayed culture conversion, poor outcome, and infection transmission [14]. In this study, in spite of the difference of cavity diameter between the two groups, the lesion diameter exhibited no statistical difference, suggesting that the lesion range was comparable between the two groups. The drug resistant tubercle bacilli will promote the cavitary disease chronically [15].

Surgical resection has been considered as an effective assistant in PTB therapy, especially in DR-TB [16, 17]. The principle of resection is that removing actively replicating bacilli obviously decreases the total organism burden in the lung. It was reported that thick-wall cavity of destroyed lung has up to 10^7^ to 10^9^ organisms even in patients with culture-negative sputum [18, 19]. Since they exposed less to host defense and anti-TB drugs, the cavitary lesions might be easy to develop drug resistance [20]. Surgical resection may improve the outcome of treatment, prevent disease progress, and reduce the probability of drug resistance. For surgical patterns, it was traditionally accepted that lobectomy or pneumonectomy was recommended in view of completely removing the lesion [21]. However, with development of anti-TB chemotherapy recent years and experienced long time of our observation, we found that sublobectomy might also be considered for localized cavitary PTB, under this background we conducted this study. The results indicated that most of the patients received operation were cured with postoperative chemotherapy except two cases lost follow-up, patients with cavity could benefit from both lobectomy and sublobectomy to prevent irreversible parenchymal damage. For drug resistant TB, a meta-analysis consist of 9153 patients indicated that patients who underwent partial lung resection had statistically significantly higher rates of treatment success [22]. Moreover, those patients who underwent pneumonectomy failed to exhibit better outcomes than those who did not undergo surgery. Prognosis seemed to be better in partial lung resection group after culture conversion, while this effect was not observed in patients who underwent pneumonectomy. In present results, only one case relapsed among the drug resistant TB who received lobectomy and no relapse was observed in the sublobectomy group. In spite of the outcomes could be caused by the enrollment bias, it suggested that sublobectomy is a selectable choice for drug resistant TB patients.

Following the technique development and equipment improvement, video assisted thoracoscopic (VATS) therapeutic resection, especially uniport VATS, was gradually adopted in pulmonary disease. In addition to patients with malignancies, it was also feasible in the treatment of PTB. Compared with lobectomy, the advantages of sublobectomy, such as more pulmonary function conservation and a shorter hospital stay, had been strongly recommended [23]. Appropriate surgical method selection may let the patient decrease grading of image characteristics with less blood loss, shorter operative time, and shorter hospital stay. Meanwhile, the relapse in our study was not found in sublobectomy group. Early referral and liberal use of VATS sublobectomy on suitable case might well provide substantial impact on reducing the bacterial burden without much trauma, which enhanced the opportunity of cure.

The surgical indication for cavitary PTB was still controversial. It was considered that the timing of operation would coincide with the lowest bacterial burden to optimize the outcome with the least morbidity. Traditionally, the patients received operation after half a month of medication, which may lead to the requirement of lobectomy as it was considered that the segmental bronchial stump may be destroyed by the residue tuberculosis bacillus. It was reported that 2–3 months of effective medication and sputum culture conversion may provide better outcome with a decreased risk of infectivity [24]. In our study, the patients received average 5 months of medication before surgery as it was obtained from the real world, suggesting that proper medication time for surgical intervention can obtain good outcomes and fewer complications.

Complications were the major concern for clinicians to oppose the surgery. It was reported that the main postoperative complication rate for multidrug resistant TB was between 15 and 40% [21, 25]. In this study, major complications occurred in 12.8% (26/203 patients), which was similar with the other reports. Prolonged air leaks, increased amount of drainage, and chylothorax were the major types. For air leak, dense adhesion may affect the whole lung and the pathologic lung expansion was not feasible, whereas lobectomy required more adhesion release to remove the whole lobe, which may increase the risk of air leak compared with sublobectomy. In addition, one of the main goals of the operation was to avoid bleeding and opening cavities, but not air leak. Thus, compared with bleeding, air leak was more acceptable for the surgeons. Meanwhile, the application of stapler on fissure may reduce the risk of air leak. Chylothorax is also a result of extensive adhesion or structural distortion, which may cause accidental injury. In the sublobectomy process, it may need less area of adhesion release, extradural separation, and mediastinal isolation, which may reduce the risk of these complications. Severe complications that restrained clinicians to select operation for cavitary PTB patients, such as respiratory failure, bronchopleural fistula, lung and other infections, empyema, wound bleeding and/or breakdown, and recurrent laryngeal nerve palsy, the fact that the MTB in the regional lymph may retain some bacteria and induce disease relapse was also concerned for surgical safety if choose sublobectomy. However, in this study sublobectomy preserved the vasculature around the bronchial stump, and the patients could be cured by postoperative medication instead of sustain the risk of bronchopleural fistula. Our results showed that there was no fatal complication occurred in both groups, indicating that sublobectomy was feasible in the treatment with fewer complications.

There were still some shortcomings in this study. Firstly, larger scale research is needed in the future to better resolve the drawback of case selection bias in present study. Secondly, there is still lack of indication about the cutting edge distance for PTB, we continually used the criteria of lung tumor resection as that the cutting edge distance is at least 2 cm from the lesion in this study. Postoperative pathology showed no tuberculosis found on the cutting edge of the specimen, whereas more in-depth investigation is needed to determine the specific distance of the cutting edge.

## Conclusion

In summary, surgery remains an important adjunct tool in the management of patients with cavitary PTB. Compared with lobectomy that was the conventional surgical treatment standard, under adequate anti-TB chemotherapy sublobectomy might also provide favorable results to the resectable lesions with faster recovery and fewer complications. The success of operation relies on good cooperation between TB physicians and thoracic surgeons, patient compliance in completing the postoperative anti-TB medicine, surgical experience, and careful follow-up.

## Data Availability

The datasets analyzed during the current study was available from the corresponding author on reasonable request.

## Abbreviations

PTB: Pulmonary tuberculosis
MTB: *Mycobacterium tuberculosis*
DR-TB: Drug-resistant tuberculosis
VATS: Video-associated thoracic surgery
LOS: Length of stay
